# On Vaccination

**Published:** 1802-01

**Authors:** 


### *On VACCINATION.*

While the ftrongeft teftimonials in favour of Vaccination are moftly confined to medical books, reports the moft fatal to its interefts continue daily to accumulate, and are circulated with much earneftnefs, and even apparent fatisfaction, by at leaft the fceptical, the anile, and the foolifh, uncontradicted and unchecked. Aware of this, I ventured almoft two years ago an opinion, that medical men fhould, from time to time, communicate their fentiments to the public, on this great national fubject, through the channel of the moft popular prints. I am forry to fay, that the hint has not been generally followed up. It was not, however, wholly unnoticed. The grofs mifreprefentations of the ftate of Vaccine Inoculation at Plymouth, having fpread through Cornwall, and occafioned much anxiety among the profeffional men, who had recommended, and in thofe families who had acceded to the new practice, the Phyficians and Surgeons of Plymouth, Stone-houfe and Dock, judged it it extremely neceffary to filence thefe abominable ftories, by publifhing a very ftrong Teftimonial to the merits of Vaccination, firft in the Medical Journal, and from thence in the Sherborne and Exeter provincial papers. Although I know that thefe fteps were, *for some time*, followed by the beft effects; and although nothing can be more evident than that they were undertaken by the Faculty to ferve the community, not themfelves; yet I am obliged to admit, that there were not wanting fome who were either ignorant or difaffected enough, or perhaps both, to reprefent their conduct on this occafion as the moft arrant piece of quackery, and the printed Teftimonial as an empirical advertifement. Good God! in the name of humanity and of common fenfe, what muft be the conftitution of fuch minds! Convinced that fome meafures of this fort are, even now, more called for than at any time, perhaps, fince the introduction of the Cow-pox, I defire again, by this opportunity, to invite profeffional men to join me in purfuing means fo obvioufly calculated to promote that great defideratum, *the general establishment of Vaccination for a few years in the united kingdom.* Let it be remembered, that this great national benefit *can only be effected through the immediate, concurring, and cordial exertions of medical men.* Thofe gentlemen who fee with me on this fubject, will find in me a ready correfpondence; in the mean time I will not omit now to fay, that from the time I had formed my opinions on this eventful queftion, I have, almoft daily, vaccinated fubjects of very different ages and defcriptions; that a great part of thofe have been fubfequently, and conftantly, expofed to an highly variolated atmofphere, (the cafual Small-pox having prevailed in this town, more or lefs, fometimes, and at this time, very badly, during the whole of the prefent year) and to every other means of infection from the Small-pox that prudence will fanction. I have not, however, I moft folemnly declare, feen nor heard of a fingle well-authenticated circumftance in the leaft degree tending to invalidate my opinions, or weaken my faith in the immenfe advantages derivable from the Jennerian Inoculation; on the contrary, my own experience, and all that of this neighbourhood, go directly to heighten my VENERATION, if I may be allowed to fay fo, for the DISCOVERY and DISCOVERER of this great bleffing. Let Dr. Haygarth's excellent regulations to prevent the fpread of the cafual Small-pox, be fully and generally acted on wherever this dreadful diforder makes its appearance: Let babes be regularly vaccinated in the firft months of infancy, when we know they are but little fufceptible of taking on the infection of the cafual Small-pox, or other contagious difeafes: Let all the medical men in the kingdom, concur in a firm determination, *not to inoculate with the Small-pox*, unlefs in particular fituations, which fhall be *unequivocally* underftood and agreed on, (a conduct I have long obferved, and fhall, under my *present convictions*, invariably adhere to:) Let all the circumftances of every queftionable or fpurious cafe be minutely inveftigated, and fully and in the moft public manner reported on, by an able and impartial committee appointed by general confent for that purpofe: Let the public mind be no longer diftracted by the circulation of dreadful accidents, and numerous failures, which are fo eagerly caught at, edited and improved by the ignorant and the prejudiced.

Were thefe, or propofals like thefe, generally adopted, the extinction of the Small-pox in thefe iflands would in a few years, very probably, be complete. It is difficult almoft to believe, that there fhould be a man of reflection or humanity in this country, who can contemplate, without enjoying the moft lively and grateful emotions, the probable attainment or even diftant poffibility of fuch an event. Yet are there fome, who, with every information, or every means of information before them, and every way capable of appreciating the refpective merits of Vaccination and the inoculated Small-pox, and of underftanding thofe vaft national benefits and individual comforts that, in all human probability, would refult from an exclufive adoption of the former, can deliberately and uniformly recommend the latter. Some, with a well feigned expectation of finding them, and therefore with an ingenious cruelty peculiarly their own, have fought for on the heads of children lately vaccinated, the approaching horns, and at the fame time have, with equally affected ferioufnefs, enquired if a fomething had not been noticed in their voices, imitative of the bellowing of the cow. Thefe fooleries, as fuch, though at firft fight moft truly laughable and ridiculous, leave fometimes indelible impreffions on weak minds, and fhould, therefore, be avoided; but feen in another point of view, they merit the fevereft reprehenfion. Great pains have been taken, — but I ought to make no common apology for admitting an expreffion, which though it ftrongly, and I believe, correctly conveys my meaning, favours of a pun, — great pains have been taken to de-Jenner-ate Vaccination and the Cow-pox. In fhort, were I to detail half the anecdotes which have reached me, relative to the conduct of the enemies of Vaccine Inoculation, it would be feen, that no means have been left unattempted, to intercept a moft bountiful gift of Providence, which it is certainly as much our intereft as it is our duty, to receive with equal gratitude and reverence.

Mr. Ring, in the laft Number of the Medical Journal, obferves, ferves, I am afraid very juftly, "That while unfavourable cafes are circulated *with great industry*, by *professed enemies of Vaccine Inoculation*, or *its pretended friends*, it appears to him, that the real friends of the practice, lulled into a ftate of falfe fecurity, are become rather remifs; and being convinced themfelves, imagine that the public are equally convinced. Hence they leave the field to their *more active opponents*, who *improve this favourable occasion to their own advantages*." *On this occasion*, however, I am not afhamed to fay, that it has been my employment, and my greateft pleafure, daily to furnifh my medical friends and neighbours with the vaccine medium; to fend it almoft to every part of the adjoining counties, and, I believe, to every quarter of the globe; to reply regularly to numerous correfpondents who have arifen to me from my attention to this fubject; to invite the poor, and vaccinate gratuitoufly, all thofe I have been able to convince, (I printed the enclofed note more than a year ago, but previoufly confulted with, and invited, fome medical friends, whom I moft cordially efteem, to join me; they did not, however, fee the fubject in the fame point of view, and thought then that the introduction of the practice had better be left to the operation of time and circumftance) than difintereftedly and obftinately to combat prejudices and objections whenever I met them.

Although I have not, certainly, if I may judge myfelf, made this recapitulation from any motives of vanity, yet I fhould be very glad indeed, if it could be faid with authority and with effect, to every medical man in the kingdom, "*Go thou and do likewise.*" Mr. Ring fays, "That in order to decide this queftion, if any gentleman who has infected two, ten or twenty of his own vaccine patients with variolous matter, will take the trouble of inoculating mine with the fame, I will furnifh him with at leaft a thoufand, on whom he may exercife his fkill. This offer the opponents of Vaccine Inoculation, *and its pretended friends*, ought to accept, or in decency to be filent."

I am very willing to truft the fate of Vaccination to the iffue of repeated inoculations with the moft active variolous matter, or of any other rational expofures to the fame principle, of as many of my vaccinated friends, or the boldeft and moft confident Anti-vaccinift fhall think proper to felect. The *genuine vaccine lymph* does, or does not, poffefs an abfolute preventive power againft variolous contagion. Such a power is, or is not, a law of Nature. The protection, if it affords protection, cannot be cafual, it muft be regular and determined. All our enquiries, all our analyfes, all our experience, all our knowledge of the oeconomies and analogies of Nature, confirm to us, in a language a language not to be miſtaken, that there are impreſſed on all matter, original and immutable principles. The philoſophical chymiſt, in his moſt ſubtle and abſtract reſearches to explore the conſtitution and dependencies of the moſt extraordinary productions of Nature, will find the ſame experiments (repeat them as often as he pleaſes, if he repeats them accurately) invariably followed by the ſame reſults, the ſame affinities, the ſame relative proportions, &c. &c. There is not indeed any thing lawleſs in phyſic. This remark holds equally true in the eccentric comet, and in the moſt inſignificant atom in creation. It is impious to doubt it. Let all medical men, therefore, and ſurely they need no other ſtimulus than the recollection that nearly one thouſand human beings fall victims every week, in the united kingdom, to the ravages of the Small-pox, a calculation drawn from the faireſt and moſt indiſputable data. Let medical men, therefore, by a generous and emulative co-operation, bring this important queſtion to a deciſion; let an experiment be inſtituted on a large ſcale, by medical men and others, whoſe judgment, characters, and reſponſibilities cannot be called in queſtion; let every poſſible publicity be given to it, and let the EVENT definitively eſtabliſh, or totally aboliſh, the VACCINE INOCULATION. Moſt readily, indeed, I ſhould particularly deſire to bring forward *a most dearly beloved infant*, to make one, on ſuch a great and intereſting an occaſion.

Let me anticipate:—That if to preſerve and extend the population of a ſtate, is to preſerve and extend its beſt riches; if the removal of, perhaps, the greateſt from the long catalogue of human afflictions, is the accompliſhment of an event, which directly, or indirectly, will conduce to the comforts and happineſs of every fire-ſide, hitherto within the reach of the Small-pox, then will, moſt aſſuredly, national honours and national remunerations, both from public and private ſources, await the moſt fortunate man in the world. With pride, with juſt and national pride, we boaſt a Newton and a Harvey; poſterity, (and let us too, at leaſt, with equal pride) will boaſt a JENNER.

