# Corrigenda et Addenda

**Published:** 1802-02

**Authors:** Richard Dunning

**Affiliations:** St. Aubya Street, Dock


### CORRIGENDA et ADDENDA.

Vol. vi. p. 482, l. 14, from bottom, for Camph. ʒij. read Camph. ʒſs.
Vol. vii. p. 6, l. 10, read Phyſics.

*A part of Mr. Dunning's Communications, laſt month, being by miſtake, ſent to different Editors, an omiſſion and conſequent obſcurity has crept into p.* 5, *l.* 17. *The parentheſis was intended to introduce the following Note, viz.*

The Small-pox prevail now in this town and are very fatal:—Inoculation for them, which in itſelf is ſometimes very troubleſome, and now and then deſtroys, tends directly to ſpread and perpetuate this afflicting diſeaſe, while every caſe of INOCULATED COW-POX is, perhaps, one ſtep towards the EXTINCTION of it.

From an earneſt wiſh to forward this moſt deſirable and, now in all probability, attainable event, wholly uninfluenced by any pecuniary conſiderations, and wholly diſclaiming every view to popularity, at the hazard however I am afraid and am ſorry for it, of appearing in the opinion of ſome a little over zealous, and it may be irregular; lar; I venture by dropping this paper among my friends and neighbours to call a little the public attention more immediately to the subject of the new inoculation, and, in this way to say, that I shall, at all times, experience the utmost satisfaction in inoculating, *gratuitously*, with Cow-pox, as many as I conveniently can, of those inhabitants in this town and parish whose circumstances will not enable them on this occasion to engage medical assistance:—And that they be encouraged to avail themselves of the great advantages to be derived from the new practice, I judge it proper to inform them that this easy means of prevention has been so fully ascertained to be an entire and lasting security against every possible exposure to the Small-pox, the fact has lately been publicly subscribed by the greater part of the most eminent and respectable Physicians and Surgeons in London, and by many who had at first started objections to it.

That the celebrated Dr. Pearson has observed, "That the chance of life appears to be greater during the Cow-pox Inoculation than under the ordinary circumstances of life; that he has in general found the health of sickly children mended by it, and does not know of any disease produced or excited by it."

That from this testimony, from many others nearly as strong, from my own experience, and I may add, from that of this neighbourhood, I can almost undertake to assert, that the degree of loathsomeness and distress usually attendant on a single bad case of natural, or even inoculated Small-pox, is greater than the whole amount of that which has hitherto arisen from all the cases, now more than ten thousand, of inoculated Cow-pox.

That as from the extreme mildness of this new practice no harm can possibly happen from it, it is become a custom with many, who cannot yet fully credit the efficiency of its protection against the Small-pox, to adopt it as a matter of course for their infants.

That this simple process, being little more than a local affection of the arm, seldom subjects to the least confinement or interruption in the management of a family.

That this disorder, if it can be called disorder, is not at all infectious, and that therefore infants at the breast, and their nurses, may be inoculated immediately in succession, or even at the same time, with little or no inconvenience to each other.

N. B. It is a confirmed observation that infants in the first months are but little liable to take on the action of the natural Small-pox, how much soever exposed to them; were they therefore, at this early period, regularly inoculated with Cow-pox, and inoculation for the Small-pox generally discontinued, (the great and obvious propriety of attending to both these circumstances will soon, I hope, be allowed) it would, by no means, be unreasonable to conclude, that the latter, having no longer but few subjects for its ravages, must necessarily in a few years disappear, and both inoculations, as far as relates to the Small-pox, consequently be superseded.

*St. Aubyn Street, Dock, Sept.* 1800. RICHARD DUNNING.

*Our Readers are therefore requested, in reading that page, to omit the parenthesis, and immediately after it, for* than *read* and.

